# Result of Some Observations Made by Benjamin Rush, M. D. Professor of Chemistry in the University of Philadelphia, during His Attendance as Physician General of the Military Hospitals of the United States in the Late War

**Published:** 1786

**Authors:** Benjamin Rush

**Affiliations:** Philadelphia


*in the University of Philadelphia, during his Attendance as Physician General of the Military Hospitals of the United States in the late War. Communicated in a Letter to Mr.* Thomas Henry, *F. R. S. &c.* — Extracted from *Memoirs of the Literary and Philosophical Society of Manchester,* Vol. II. 8vo. Cadell, *London,* 1785.

*To Mr.* THOMAS HENRY.

Dear Sir,

THE inclosed observations are at your service. Instead of dilating them with theories and cases, which would add only to the number of books, but not to the stock of facts, I send them to you in as short a compass as possible. They are not so fit for the public eye as I could wish; but if you think them worthy of a place in your transactions, you are welcome to them.

Be assured, dear Sir, of the great regard of

Your friend and humble servant,

BENJAMIN RUSH.

*Philadelphia,*
July, 22, 1785.

### Result *of* Observations, *&c.*

1. The principal diseases were putrid fevers. Men, who came into the hospitals with pleurisies, rheumatisms, &c. soon lost the types of their original diseases, and suffered, or died, with the putrid fever.

2. This putrid fever was often artificial, produced by the want of sufficient room and cleanliness.

3. It always prevailed most, and with the worst symptoms, in winter: a free air, which could only be obtained in summer, always prevented or checked it.

4. Soldiers billited in private houses, escaped it, and generally recovered soonest from all their diseases.

5. Convalescents, and drunken soldiers, were most exposed to putrid fevers.

6. The remedies that appeared to do most service in this disease, were tartar emetic in the beginning, gentle doses of laxative salts, bark, wine, (two or three bottles a day in many cases) and sal volatile.

7. In all those cases where the contagion was received, cold seldom failed to render it active. Whenever an hospital was removed in winter, one one half of the patients generally ſickened in the way, or ſoon after their arrival at the place to which they were ſent.

8. The army, when it lay in tents, was always more ſickly, than when it lay in the open air: it was always more healthy, when kept in motion, than when it lay in an encampment.

9. Militia officers, and ſoldiers, who enjoyed health during a campaign, were often ſeized with fevers upon their return to the *Vita Mollis*, at their reſpective homes. There was one inſtance of a militia captain, who was ſeized with convulſions the firſt night he lay on a feather bed, after lying ſeveral months on a matraſs and on the ground. The fever was produced by the ſudden change in the manner of ſleeping, living, &c. It was prevented, in many caſes, by the perſon lying, for a few nights after his return to his family, on a blanket before the fire.

10. I met with ſeveral inſtances of bubos, and ulcers in the throat, as deſcribed by Dr. Don. Monro: they were miſtaken by ſome of the junior ſurgeons for venereal ſores, but they yielded to the common remedies of putrid fevers.

11. Thoſe patients in putrid fevers, who had large ulcers, and even mortifications on their backs or limbs, generally recovered.

12. There were many inſtances of patients in putrid fevers who, without any apparent ſymptoms of diſſolution, ſuddenly fell down dead, upon being moved; this was more eſpecially the caſe, when they aroſe to go to ſtool.

13. Thoſe officers who wore flannel ſhirts, or waiſtcoats next to their ſkin, in general eſcaped fevers, and diſeaſes of all kinds.

14. Lads under twenty years of age, were ſubject to the greateſt number of camp diſeaſes.

15. The ſouthern troops were more ſickly than the northern or eaſtern troops.

16. The native Americans were more ſickly than the Europeans.

17. Men above thirty and thirty-five years of age were the hardieſt ſoldiers in the army. Perhaps this was the reaſon why the Europeans were more healthy than the native Americans; they were more advanced in life.

18. The troops from Maryland, Virginia, and North Carolina, ſickened for the want of ſalt proviſions. Their ſtrength and ſpirits were only to be reſtored to them by means of ſalt bacon. I once ſaw a private in a Virginia regiment giment throw away his ration of choice fresh beef, and give seven shillings and six-pence specie for a pound of salt meat.

19. Most of the sufferings, and mortality in our hospitals, were occasioned not so much by actual want or scarcity of any thing, as by the ignorance, negligence, &c. in providing necessaries for them. After the *purveying* and *directing* departments were separated (agreeably to the advice of Dr. Monro) in the year 1778, very few of the American army died in our hospitals.

